# Analyzing the impact of fiscal conditions on private health expenditures in OECD countries: a quantile ARDL investigation

**DOI:** 10.1007/s10754-024-09377-0

**Published:** 2024-05-22

**Authors:** Nuno Silva, Aida Isabel Tavares, Matheus Koengkan, José Alberto Fuinhas

**Affiliations:** 1 Faculty of Economics, CeBER, University of Coimbra, Av Dias da Silva 165, 3004-512 Coimbra, Portugal; 2 CEISUC - Centre for Health Studies and Research, University of Coimbra, Coimbra, Portugal; 3 ISEG, UL - Lisbon School of Economics and Management, University of Lisbon, Lisbon, Portugal; 4 University of Coimbra Institute for Legal Research (UCILeR), University of Coimbra, 3000-018 Coimbra, Portugal

**Keywords:** Private health expenditures, Public fiscal conditions, Universal health coverage, OECD countries, C33, H51, H62, H63, I11, I18

## Abstract

Organization for Economic Co-operation and Development (OECD) countries have embraced the aim of universal health coverage, as established in Sustainable Development Goal (SDG) 3.8. This goal guarantees access to quality healthcare services without financial hardship or poverty. Additionally, it requires correct and adequate financing sources. A country with weak protection for its population tends to spend less on healthcare and experiences a high share of out-of-pocket payments (OOPs), increasing the likelihood of people falling into poverty. This study aims to understand the relationship and causal effects between macroeconomic and public fiscal conditions and private health expenditure in OECD countries between 1995 and 2019. We retrieved OECD data for 26 OECD countries for the period 1995–2019. Panel AutoRegressive Distributed Lag (PARDL) and panel quantile AutoRegressive Distributed Lag (PQARDL) models were estimated to examine the relationship between private health expenditures and macroeconomic and public fiscal variables. Our results reveal a positive influence of government debt and economic freedom on private health expenditures. They also show a negative influence of the government budget balance, government health expenditures, and economic growth on private health expenditures. These results collectively suggest that public fiscal conditions will likely impact private health expenditures. The findings of this study raise concerns about the equity and financial protection objectives of universal health coverage in OECD countries.

## Introduction

Universal health coverage (UHC) has been established as a Sustainable Development Goal (SDG 3.8) so that people can access quality healthcare services without facing financial hardship and falling into poverty. The achievement of this goal requires a correct and adequate financing source. Health systems are financed through two general forms of financial resource collection: compulsory and non-compulsory. The compulsory forms include tax-based and social insurance schemes requiring public expenditures. The non-compulsory forms include out-of-pocket payments (OOPs) and voluntary health insurance (VHI), which households pay for to obtain health care. These private healthcare expenditures raise significant equity concerns, as some families may incur catastrophic health expenses and fall into poverty. For this reason, a well-designed compulsory financing structure is the best way to guarantee the path towards UHC and protect people from financial hardship. A country that provides weak protection for its population tends to spend less on health care and register a high share of OOPs, which increases the likelihood of people falling into poverty.

Macroeconomic and fiscal conditions influence how health systems are financed, specifically public and private health expenditures. For instance, the global financial crisis and the International Monetary Fund (IMF) interventions in different countries toward fiscal consolidation resulted in a decrease in public health expenditures. However, research on the relationship between macroeconomic and fiscal conditions and private health expenditures is underexplored, despite some descriptive analyses and statistical inferences presented in some documents (WHO, 2020, 2021; OECD, 2015; Thomson et al., 2019) due to its importance in assessing universal health coverage and protecting people from financial hardship.

The existing literature has provided a limited exploration of the relationship between macroeconomic and fiscal conditions and private health expenditures. The literature lacks comprehensive understanding, which challenges any analysis on this topic and may hamper the assessment of universal health coverage and protection from financial hardship. This investigation addresses this gap and aims to answer the research question: What is the relationship between private health expenditures and fiscal conditions in Organization for Economic Co-operation and Development (OECD) countries? This research question raises several general testable hypotheses. First, quest the substitutive relationship between public and private health expenditures and its potential difference across the distribution of the different levels of private health expenditures in different countries. Second, it shows the effect of public finance and fiscal indicators, such as public debt and public budget balance, on private health expenditures. And third, the impact of economic growth and the associated economic freedom on private health expenditures.

This empirical investigation will use the PARDL (Panel Autoregressive Distributed Lag) and PQARDL (Panel Quantile Autoregressive Distributed Lag) models to address the research question and test the hypotheses. These models will be applied to a panel dataset of 26 OECD countries, covering 1995 to 2019. We will use these estimates to analyze the relationship between private health expenditures and public fiscal conditions.

This research aims to identify a suitable financing source for achieving universal health coverage (UHC) in OECD countries. It addresses the relationship between private health expenditures and macroeconomic and fiscal variables specific to these countries, providing insights into the financial sustainability of their health systems and protecting individuals from financial hardship.

The findings will contribute to evidence-based policies tailored to OECD countries, ensuring equitable access to quality healthcare without pushing individuals into poverty. The study takes a comprehensive approach, considering various macroeconomic and fiscal variables specific to OECD countries, and includes economic freedom indicators to explore the influence of economic policies and regulatory frameworks.

**Fig. 1:**
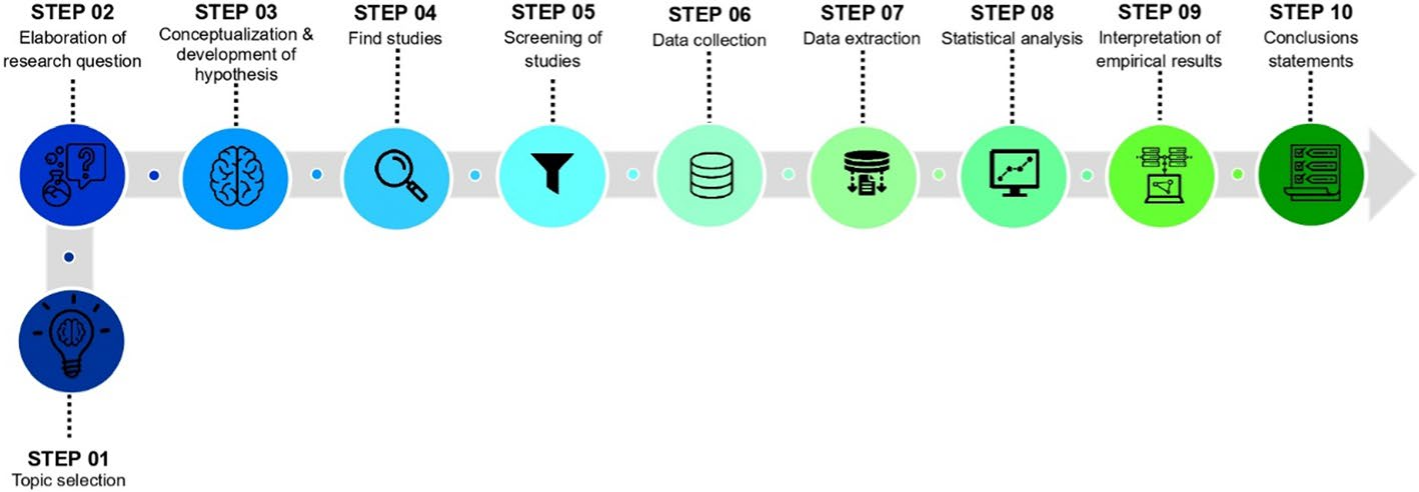
Scientific method steps. The authors created this figure

This study's unique contribution lies in exploring the relationship between private health expenditures and macroeconomic and fiscal variables within the context of universal health coverage in OECD countries. Filling an underexplored research gap expands the knowledge base on the financial dynamics of healthcare systems in OECD countries. The findings will guide policymakers in designing effective financing structures and resource allocation for sustainable and equitable health systems that protect individuals from financial hardship. This study adopted a systematic approach to ensure clarity, coherence, and a well-structured investigation by adhering to the steps of the scientific method, as illustrated in Fig. 1 below.

The organization of this research is as follows. The next section provides a comprehensive summary of the relevant literature, serving as a foundation for the subsequent sections. "Background and the previous literature perspective" section presents the background and perspective of previous literature, and "Hypotheses description" section describes the hypothesis of this investigation. "Methods" section outlines the research methodology and data sources utilized, while "Results" section presents a detailed analysis of the empirical results. The main findings derived from the analysis are thoroughly examined and discussed in "Discussion" section. Lastly, "Conclusion" section offers valuable policy implications derived from the study's findings, providing a conclusive perspective.

## Background and the previous literature perspective

Universal Health Coverage (UHC) has its roots in the post-Second World War era when health as a right was stated in the WHO Constitution (WHO, 1948) and in the Universal Declaration of Human Rights (UN, 1948). The importance of the concept grew in 2010 when the World Health Report was published (WHO, 2010). UHC aims to ensure that people can access quality healthcare services, prevent public health risks, and protect individuals from financial risks due to illness (Maeda et al., 2014). The relevance of UHC was emphasized by turning it into one SDG, specifically SDG 3.8 (WHO, 2020), which includes financial risk protection and access to quality essential healthcare services. Achieving UHC is not a static goal but a path towards UHC (Kutzin et al., 2016; WHO, 2010). This path requires financing health systems to provide access to healthcare and prevent financial hardships for people using health services.

UHC financing sources can be divided into compulsory schemes and non-compulsory payments. The first category encompasses government or tax-financed schemes, social health insurance (SHI), and compulsory private health insurance. The difference between SHI and tax-financed schemes is meaningless nowadays because, if compulsion and subsidization (the Fuchs conditions) hold, the movement towards UHC is guaranteed (Kutzin et al., 2016). What matters for a health financing policy is how well UHC protects people from catastrophic expenditures and unmet health needs (WHO, 2021).

Non-compulsory payments include two general categories: voluntary health insurance and out-of-pocket payments, usually called private health expenditures. Voluntary health insurance implies that people pay a premium to access an insurance policy not mandated by the government; it is a personal choice. Out-of-pocket payments (OOPs) refer to household spending on health, which may include consultation fees, payments for medicines or other medical supplies, laboratory tests, and hospitalization costs (OECD, 2011; Thomson et al., 2019).

Out-of-pocket payments are considered the most regressive form of health system financing, imposing a high financial burden on family budgets that may result in catastrophic expenditures and poverty. There is a strong positive correlation between the incidence of catastrophic expenditures and the level of OOPs paid by families despite the high variability of OOPs and catastrophic expenditures across countries (Thomson et al., 2019). OOPs measure well-being (Doorslaer et al., 2006) and are often used as a proxy for living standards (Cavagnero & Bilger, 2010). In countries where health systems provide weak financial protection to people, OOPs that lead to catastrophic expenditures mainly arise from expenditures on outpatient medicines and inpatient care. On the other hand, in countries with better financial protection, catastrophic expenditures are mainly due to dental and inpatient care (Thomson et al., 2019). Cross-country analysis suggests that when the OOPs level is below 15% of total health expenditure, few households will likely face catastrophic expenditures (WHO, 2021; Xu et al., 2003). In comparison, for OOPs above 29% of total health expenditure, the likelihood of increasing the poverty level in the country is high (Sirag & Mohamed Nor, 2021).

A negative relationship between non-compulsory payments and public health expenditure has been established (Thomson et al., 2019), especially between OOPs and public health expenditure (Grigorakis et al., 2018; Kutzin et al., 2016; WHO, 2021; Xu et al., 2003). A study published in 2012 estimated that an increase of 1 percentage point in the OOPs' share of total health expenditure was associated with a decrease of 1.5–3.4% in public health expenditure per capita (Cylus et al., 2012). This implies that OOPs can be reduced by increasing public expenditure on health, and some authors found that for a given public health expenditure to gross domestic product (GDP), the share of OOPs in total health spending decreases (Jowett et al., 2016). However, there is considerable variability and differences across countries regarding the relationship between OOPs and health expenditures, and these differences may not wholly explain the different levels of catastrophic expenditures (Thomson et al., 2019).

Despite the scarcity of literature concerning the factors associated with private health expenditure, general results link health expenditures to economic growth (Beylik et al., 2022; Hitiris & Posnett, 1992; Ozyilmaz et al., 2022; Samadi & Homaie, 2013). While some studies find an inelastic relationship (Beylik et al., 2022; Samadi & Homaie, 2013) between those variables, others find an elastic relation between health expenditures and economic growth (Ozyilmaz et al., 2022). Additional evidence shows economic growth associated with economic freedom (Brkić, 2020; Haan & Sturm, 2000; Scully, 2002). Thus, to some extent, it may be inferred that private health expenditure is related to economic freedom (Stroup, 2007) because of the open possibility that the market may respond to unsatisfied or unmet demand for health services. However, despite the potential improvement in well-being related to this economic freedom (Stroup, 2007), private provision of healthcare services may raise equity concerns and endanger UHC.

Health expenditure growth has been above GDP growth in most OECD countries, and approximately 3/4 of this expenditure is public (OECD, 2015). Since the global financial crisis, there has been a slowdown in the growth of health spending and even substantial reductions due to austerity policies in countries such as Greece, Ireland, Italy, Portugal, and Spain. These reductions in public expenditure on health have resulted in negative impacts on people's health, OOPs, and catastrophic expenditures (Mladovsky et al., 2012; Palasca & Jaba, 2015), to the extent that the IMF recently began to support an increase in public spending, often temporarily. Nevertheless, the IMF continues to stand for fiscal consolidation and the reduction of public debt in most countries around the World (Razavi et al., 2021).

Little research exists analyzing the determinants of private health expenditure (Fan & Savedoff, 2014), and even less analyzing the influence of macroeconomic and public fiscal conditions of a country on household health expenditures. In general, the findings point to some effects of macroeconomic and fiscal conditions on the level of OOPs and private medical expenditures across countries. Table 1 below summarizes the empirical studies that explain OOP and private spending across countries.

**Table 1:** Summary of previous studies on factors explaining private health expenditure

Study	Countries	Period	Independent variables	Method	Main findings
Musgrove et al. (2002)	191 WHO countries	1997	GDP per capita	Linear regression	Negative association between GDP per capita and OOPs
Clement et al. (2004)	22 OECD countries	1960–1997	GDP per capita	Cointegration approach	Private medical consumption seems to be luxurious good
Xu et al. (2011)	143 countries	1995–2008	GDP per capita, government expenditures, health public expenditures, age structure, health system financing characteristics, diseases patterns	Fixed effects and dynamic panel data	Government health expenditure and OOPs follow different paths
Fan and Savedoff (2014)	126 countries	1995–2009	GDP per capita, government expenditures, age structure	Fixed effect panel data	Declining shares OOPs result from political movements and social change; OOPs as a share of total health spending is not related to income
Keegan et al. (2013)	27 countries	2007–2009	GDP, unemployment, government debt	Construct a recession severity index and estimate a linear regression	Negative relationship between recession severity and OOPs growth
Grigorakis et al. (2018)	26 OECD countries	1995–2013	GDP per capita, government expenditures, government debt, unemployment rate, inflation rate	GMM dynamic panel data	Negative effect of public expenditure on OOPs; unemployment rate increases OOPs

This table was created by the authors

There appears to be an empirical relationship between OOPs, private health expenditures, and a country's macroeconomic and fiscal conditions. Furthermore, there is a potential conflict between decisions regarding health expenditures and fiscal policies aimed at fiscal consolidation.

## Hypotheses description

After describing previous studies, according to those publishing findings, the main testable hypotheses of this investigation may be postulated as follows.

**H1**: There is substitutability between public and private health goods and services expenditures.

**h1.1**: Household private expenditures on healthcare goods and services (% GDP) and final government expenditures on healthcare goods and services plus compulsory healthcare schemes (% GDP) are substitutes (Grigorakis et al., 2018; Kutzin et al., 2016; Thomson et al., 2019; WHO, 2021; Xu et al., 2003).

**h1.2**: Conditional on h1.1, the substitutability of the public by private health expenditures should be more pronounced for low levels (quantiles) of private expenditure in healthcare goods and services. This state supports the idea that private health services meet a specific demand, and households are reluctant to switch to public health services (Sirag & Mohamed Nor, 2021; WHO, 2021; Xu et al., 2003).

**h1.3**: Conditional on h1.1, the substitutability of public by private health expenditures should be more pronounced for high private household spending levels on healthcare goods and services. This situation indicates that private expenditures in healthcare are more of a burden than an option for households (Sirag & Mohamed Nor, 2021; WHO, 2021; Xu et al., 2003).

**H2**: The stance of public finances impacts the provision of private health services and, consequently, private healthcare expenditures.

**h2.1**: High levels of general government debt (% GDP) hamper public health services, forcing households to rely on private health services by increasing private healthcare expenditures (Mladovsky et al., 2012; Palasca & Jaba, 2015; Razavi et al., 2021).

**h2.2**: Budget consolidation may have two opposite effects on private health expenditure: (i) the reduction in public expenditure leads to a deterioration in the quality of public health services, which prompts families to utilize private health services and increase private health expenditure; (ii) budget consolidation reduces families' disposable income due to tax increases, leading them to forego some health expenditures, thus reducing private healthcare expenditure (Razavi et al., 2021).

**H3**: Economic growth improves public finances, enabling public health services by increasing public expenditure and decreasing private spending on health services as a percentage of GDP.

**h3.1**: Private health expenditures are income-inelastic relative to economic growth (Beylik et al., 2022; Hitiris & Posnett, 1992; Ozyilmaz et al., 2022; Samadi & Homaie, 2013).

**H4**: Higher economic freedom implies that households may rely more on the wide availability of private health services and increase private health expenditure (Brkić, 2020; Haan & Sturm, 2000; Scully, 2002; Stroup, 2007).

## Methods

**Fig. 2:**
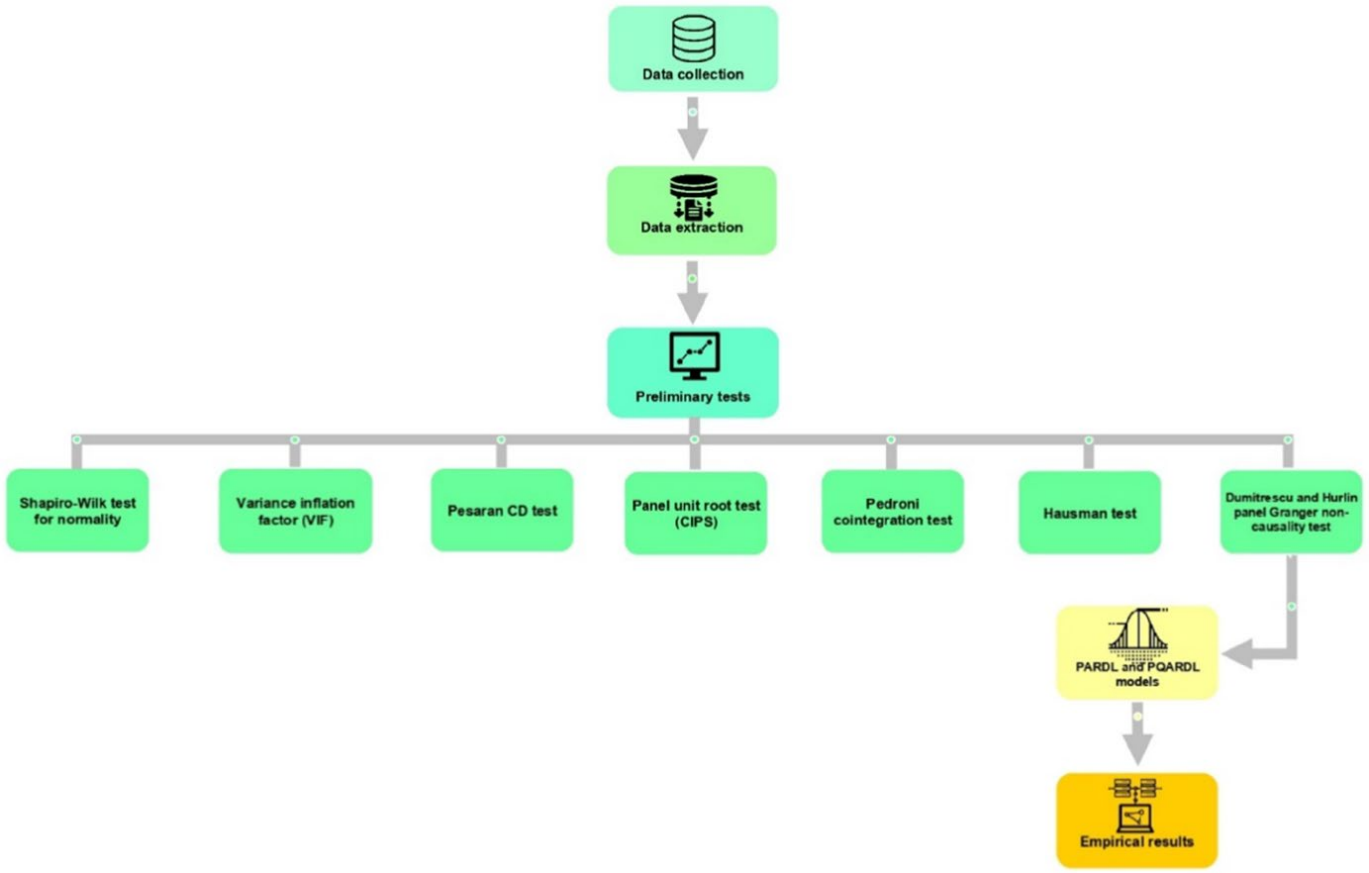
Methodological framework. The authors created this figure

This section provides a comprehensive overview of this research study's data sources and methodology. Figure 2 below shows the methodological framework that this investigation will follow.

### Data

This study centers on private health within twenty-six OECD European countries from 1995 to 2019. The selection of this group of countries is motivated by their uniform characteristics, given that most of these nations share a consistent cultural foundation, possess comparable levels of economic advancement, and uphold universal healthcare coverage. The commencement of this research in 1995 is due to it being the earliest year with accessible data, while the conclusion in 2019 aims to circumvent the disruptive COVID-19 years.

This investigation employs several explanatory variables to assess different factors that may affect households' health expenditures: (i) General government debt and general government balance aim to evaluate the impact of fiscal and budgetary policies on households' willingness to spend on healthcare; (ii) Government spending on healthcare is used to assess the relationship, whether it is characterized by substitutability or complementarity, between private and public healthcare expenditures; (iii) Economic growth indicates whether private healthcare spending is income elastic or inelastic; (iv) Economic freedom influences the provision of private healthcare services. Table 2 below displays the variables used, their sources, and a brief description.

**Table 2:** Data description

Variables	Description	Source
Priv_Exp	Private health expenditures (% GDP). It includes both household out-of-pocket payments and voluntary healthcare payment schemes	OECD (2023)
Ec_Growth	Percentage growth rate of the GDP per capita (constant 2015 USD)	WBD (2023)
Gov_Exp	Government health expenditures and compulsory healthcare schemes (% GDP)	OECD (2023)
GG_balance	General government balance as a percentage of GDP	OECD (2023)
GG_debt	General government debt as a percentage of GDP	OECD (2023)
Ec_Freedom	The Index of Economic Freedom assesses the control of individuals of his/her own labor and property (scale 0 to 100). It is based on 12 quantitative and qualitative factors grouped in four broad categories: rule of law, government size, regulatory efficiency, and open markets	Heritage Foundation (2023)

This table was created by the authors

Table 3 below presents the descriptive statistics. On average, public health spending is three times higher than private spending, and the standard deviation of the former is more than twice that of the latter. Private health expenditure exhibits a wide range, with the maximum being almost ten times higher than the minimum. General government debt also displays considerable variation across countries, ranging from nearly zero for Estonia in 2001 to two times the GDP value for Greece in 2019. The same pattern is observed for the general government balance and economic growth. The 2008 financial crisis resulted in shallow values of − 32.1188% of GDP for the general government balance and − 14.4643% of GDP per capita for economic growth. In contrast, the Economic Freedom Index shows more homogeneity, with a mean value of approximately 69 and a standard deviation of less than one-tenth of the average.

**Table 3:** Descriptive statistics

Variables	Obs.	Mean	Std.-Dev.	Min	Max
Priv_Exp	629	2.0095	0.7185	0.3980	3.9180
Ec_Growth	624	2.2539	3.3420	− 14.4643	23.2009
Gov_Exp	629	6.1701	1.4934	2.7300	9.8270
GG_Balance	647	− 1.8631	4.3629	− 32.1188	18.6378
GG_Debt	629	67.0594	36.0187	6.6537	200.7501
Ec_Freedom	638	68.9554	6.5466	49.7000	82.6000

This table was built using the Stata function *summarize*. Obs. denotes the number of observations; Std.-Dev. denotes the standard deviation; Min. and Max. denote minimum and maximum, respectively

### PARDL and PQARDL

One of the main advantages of PARDL is its ability to decompose the total effects of the covariates on the dependent variable into their short-run and long-run parts. Thus, it accounts for delays in the response of private healthcare spending to changes in the explanatory variables. Furthermore, this method is robust to variable endogeneity and allows us to consider stationary and integrated order one variables together. The PARDL model may be specified as follows:

1 $$DPriv\_Exp_{i,t} = \alpha_{i} + \gamma Priv\_Exp_{i,t - 1} + \mathop \sum \limits_{j = 1}^{5} \beta_{j} DX_{i,t}^{j} + \mathop \sum \limits_{j = 1}^{5} \theta_{j} X_{i,t - 1}^{j} + \varepsilon_{i,t} ,$$

where $X_{i,t}^{j}$ represents the value of the explanatory variable j for country i in year t, $X = \left\{ {Gov\_Exp,GG\_Debt, GG\_Balance,Ec\_Growth,Ec\_Freedom} \right\}$, “*D*” is the first difference operator, and $\varepsilon_{i,t}$ is the error term for country i in year t. In this model, the short-run effects of the covariates are given by the $\beta_{j}$ estimates, while the long-run effect of covariate j can be computed as $- \theta_{j} / \gamma$.

Despite its many advantages, this method also has shortcomings: it only focuses on the effects of the covariates on the conditional mean of the dependent variable, is sensitive to outliers, and assumes normally distributed data. The Quantile Autoregressive Distributed Lag model (Cho et al., 2015) overcomes these limitations, as it can assess the impact of the covariates on all the support of the dependent variable conditional distribution. Thus, it is suitable to assess the differing effects of the explanatory variables on countries with low and high private health expenditures. Furthermore, quantile regression does not require normally distributed data and is broadly insensitive to outliers. This investigation represents the PQARDL model for quantile $\tau$ by Eq. (2) below:

2 $$DPriv\_Exp_{i,t} = \alpha_{i} \left( \tau \right) + \gamma \left( \tau \right)LPriv\_Exp_{i,t - 1} + \mathop \sum \limits_{j = 1}^{5} \beta_{j} \left( \tau \right)DX_{i,t}^{j} + \mathop \sum \limits_{j = 1}^{5} \theta_{j} \left( \tau \right)X_{i,t - 1}^{j} + U_{i,t} ,$$

where $U_{i,t} = Priv\_Exp_{i,t} - Q_{Priv\_Exp} \left( {\tau |Priv\_Exp} \right)$, and $Q_{Priv\_Exp} \left( {\tau |\Im_{t - 1} } \right)$ represents the $\tau$-quantile of the conditional distribution of $Priv\_Exp$ given the information available at year t − 1 $\left( {\Im_{t - 1} } \right)$. Note that this model allows for different effects of the covariates on private healthcare spending on different quantiles of its conditional distribution support.

### Dumitrescu and Hurlin panel Granger non-causality test

Traditional Granger causality tests assess the time series interdependence between variables. When applied to panel data, they test whether a variable Granger-causes another one everywhere in the panel. This hypothesis is too restrictive, as it ignores panel heterogeneity of the causal relationships and regression model. The Granger non-causality test proposed by Dumitrescu and Hurlin (2012) is robust to both these features. It is based on the cross-section mean of the Wald statistics of standard Granger non-causality tests.

To apply the test, we estimate the following regression for each country:

3 $$y_{i,t} = \alpha_{i} \left( \tau \right) + \mathop \sum \limits_{p = 1}^{P} \gamma_{i}^{p} y_{i,t - p} + \mathop \sum \limits_{p = 1}^{P} \beta_{i}^{p} x_{i,t - p} + \upsilon_{i,t} ,$$

where $\upsilon_{i,t}$ is the error term for country i in year t, and P represents the number of lags. Then, we compute individual Wald statistics for the null hypothesis that the variable $x_{i}$ does not Granger-cause $y_{i}$, $\beta_{i}^{\left( p \right)} = 0$, for $p = 1, \ldots ,P$. (Dumitrescu & Hurlin, 2012) suggest various ways to test the null hypothesis that $x_{i}$ does not Granger cause $y_{i}$, against the alternative that it does cause it for at least one country. Of those, we choose $\tilde{Z}_{N}^{Hnc}$ because its size is the closest one to the nominal size for small samples. This investigation computes the value of this statistic as follows:

4 $$\tilde{Z}_{N}^{Hnc} = \sqrt {\frac{N}{2 \times P} \times \frac{{\left( {T - 2P - 5} \right)}}{{\left( {T - P - 2} \right)}}} \times \left[ {\frac{{\left( {T - 2P - 3} \right)}}{{\left( {T - 2P - 1} \right)}}W_{N,T}^{Hnc} - P} \right],$$

where *N* represents the number of countries, T is the number of years, and $W_{N,T}^{Hnc}$ is the cross-sectional average of the individual Wald tests. This statistic converges in distribution to a standard normal as $N \to \infty$.

### Preliminary tests

Before estimating the models, it is essential to carry out several tests to assess the properties of the data and to select the most appropriate estimator:

(i) Shapiro–Wilk normality test (Royston, 1983). According to the null hypothesis, the data distribution is normal. Non-normal data supports the choice of using quantile regression in this investigation.
(ii) Variance inflation factor (VIF) examines whether regressors are multicollinear, leading to unreliable estimates.
(iii) Cross-sectional dependence (CSD) test (Pesaran, 2021). This test assesses the variables' cross-sectional dependence in the panel of countries. The null hypothesis of this test assumes cross-sectional independence.
(iv) Pesaran panel unit root test (Pesaran, 2007). This test checks if the variables are stationary. The null hypothesis assumes non-stationarity.
(v) Cointegration test (Pedroni, 1999). The null hypothesis of this test states that the variables are not cointegrated. Using non-stationary variables in a regression context may result in statistically significant estimates that do not reflect a true relationship between the variables—spurious regressions. This problem can be avoided when the variables under consideration are cointegrated, i.e., if a linear combination of the variables is stationary. Cointegration avoids the spurious regression problem and renders parameter estimates super-consistent.
(vi) Hausman test (Hausman, 1978). This test relies on the comparison of fixed and random effects estimates. If the null hypothesis holds, both estimators are consistent, and this empirical investigation should use random effects as it is an efficient estimator.

## Results

This section begins by providing the results of the preliminary tests, followed by the application of Dumitrescu and Hurlin's (2012) Granger non-causality tests. Lastly, this section presents the estimates obtained through PARDL and PQARDL methods.

Table 4 below displays the outcomes of the Shapiro–Wilk test for normality, where the null hypothesis assumes that the data adheres to a normal distribution. The test results strongly reject this hypothesis for all variables. This rejection reinforces the decision of this investigation to employ quantile regression as the estimation method, as it exhibits robustness to the distribution of the data.

**Table 4:** Shapiro–Wilk test for normality

Variables	Obs.	W	V	Z	Prob > z
Priv_Exp	629	0.969	12.868	6.204	0.0000***
Gov_Exp	629	0.987	5.390	4.091	0.0000***
GG_Debt	629	0.955	18.421	7.075	0.0000***
GG_Balance	647	0.918	35.026	8.646	0.0000**
Ec_Growth	624	0.922	32.085	8.420	0.0000
Ec_Freedom	638	0.990	4.388	3.594	0.0002

The null hypothesis of the Shapiro–Wilk test assumes that the data is normally distributed. ***, and ** denote statistical significance at 1%, and 5%, levels, respectively

The Variance Inflation Factor test (VIF) results are presented in Table 5 below. The VIF values for all variables are below ten, and the mean VIF is significantly below the commonly accepted threshold of six. Hence, there is no need for concern regarding multicollinearity during the estimation of the models.

**Table 5:** Variance inflation factor (VIF)

Variables	VIF	1/VIF	Mean VIF
Gov_Exp	1.52	0.658	
GG_Debt	1.67	0.598	
GG_Balance	1.26	0.794	1.45
Ec_Growth	1.30	0.770	
Ec_Freedom	1.48	0.677	

The results of the Pesaran (2021) cross-sectional dependence test are presented in Table 6 below. The null hypothesis of cross-sectional independence is strongly rejected in all cases, indicating cross-country dependence, a common characteristic of macro-panels. It is important to note that the standard errors of fixed effects estimators can be biased in the presence of cross-sectional dependence. Therefore, this investigation mitigates this bias using Driscoll and Kraay's (1998) standard errors.

**Table 6:** Pesaran CD test

Variables	CD-test	*p* value	Corr	Abs (corr)
Priv_Exp	17.222	0.000	0.20	0.47
Gov_Exp	41.252	0.000	0.47	0.61
GG_Debt	37.003	0.000	0.43	0.63
GG_Balance	32.518	0.000	0.36	0.40
Ec_Growth	54.497	0.000	0.62	0.62
Ec_Freedom	32.185	0.000	0.36	0.46

The null hypothesis of the Pesaran CD test assumes cross-sectional independence

The results of the panel unit root tests, presented in Table 7 below, provide clear evidence regarding the stationarity of the variables. Specifically, the variables GG_Balance and Ec_Growth are found to be stationary, indicating that they exhibit stable long-term behavior. On the other hand, the variable GG_Debt is determined to be non-stationary, suggesting that it has a trend component. When examining the hypothesis of non-stationarity, it is rejected for the variables Priv_Exp and Gov_Exp in the specification without a trend. However, in the specification with a trend, the null hypothesis of non-stationarity is not rejected. Furthermore, all the variables exhibit stationarity when considered in their first differences. This suggests that taking the differences between consecutive observations removes long-term trends and renders the variables stationary.

**Table 7:** Panel unit root test (CIPS)

Panel unit root test (CIPS)					
Variables in levels	(Zt-bar)		Variables in first differences	(Zt-bar)	
	No trend	Trend		No trend	Trend
Priv_Exp	− 1.871**	− 0.722	D_Priv_Exp	− 11.877***	9.958***
Gov_Exp	− 1.402*	2.032	D_Gov_Exp	− 11.110***	− 9.431***
GG_Debt	0.116	1.151	D_GG_Debt	− 9.714***	− 8.214***
GG_Balance	− 2.977***	− 2.904***	D_GG_Balance	− 16.885***	− 14.357***
Ec_Growth	− 5.930***	− 4.191***	D_Ec_Growth	− 16.200***	− 13.980***
Ec_Freedom	− 1.692**	0.143	D_Ec_Freedom	− 13.689***	− 11.155***

"D" indicates the first difference. The null hypothesis for CIPS states the series have a unit root. ***, **, and * denote statistical significance at 1%, 5%, and 10% levels, respectively

Given the ambiguous results obtained from the panel unit root tests conducted on the dependent variable, this research proceeds to perform the Pedroni cointegration test to address the issue of spurious regression. This investigation employs both versions of the Phillips–Perron t-test to ensure robustness against heterogeneity. The results of these tests, as shown in Table 8 below, reject the null hypothesis of no cointegration.

**Table 8:** Pedroni cointegration test

Pedroni cointegration test		
Estimator	Statistic	*p* value
Modified Phillips–Perron t	4.3710	0.0000
Phillips–Perron t	− 1.6776	0.0467

The null hypothesis for the Pedroni cointegration is no cointegration, while the alternative states that all panels are cointegrated

Based on these findings, one can confidently conclude that there is a stationary linear combination between the variables under investigation. This implies a long-run relationship or equilibrium between the variables rather than just a spurious correlation.

The results of the Hausman test, as presented in Table 9 below, provide strong evidence against the null hypothesis that the random effects estimator is consistent. Therefore, this investigation uses the fixed effects estimator to estimate these models.

**Table 9:** Hausman test

Hausman test		
Test distribution	Statistic	*p* value
Chi-squared(13)	56.15	0.0000

According to the null hypothesis, the random effects estimator is consistent

The results of Dumitrescu and Hurlin's (2012) panel Granger non-causality test, presented in Table 10 below, clearly indicate that all the explanatory variables significantly influence private healthcare expenditure. Additionally, the findings provide evidence of bi-directional causality for Gov_Exp, Ec_Growth, and Ec_Freedom, suggesting a reciprocal relationship between these variables and private healthcare expenditure.

**Table 10:** Dumitrescu and Hurlin panel Granger non-causality test

Dumitrescu and Hurlin panel Granger non-causality test		
Independent variables	Dependent variable (Priv_Exp)	
	Is caused by	Causes
Gov_Exp	4.1833***	3.7264***
GG_Debt	3.5275***	8.7202***
GG_Balance	2.8652***	0.1947
Ec_Growth	2.1504**	− 0.1862
Ec_Freedom	6.4182***	2.0808**

The null hypothesis for the Dumitrescu and Hurlin test states that there is no causal relationship in any panel. ***, and ** denote statistical significance at 1%, and 5% levels, respectively

Tables 11 and 12 below provide the estimation results and the short and long-run effects of the covariates on household healthcare expenditures. In particular, Table 11 displays the estimation results of both PARDL and PQARDL models. Let us look at these tables to examine the findings in detail.

**Table 11:** Estimation results

Independent variables	Dependent variable (Priv_Exp)					
	PARDL model	PQARDL model				
		Quantiles				
	Mean	10th	25th	50th	75th	90th
D_Gov_Exp	− 0.0781	− 0.0822***	− 0.0056	0.0007	0.0195	0.0148
D_GG_Debt	0.0022**	− 0.0002	0.0010	0.0012	0.0013*	0.0032
D_GG_Balance	− 0.0056*	− 0.0061	− 0.0059***	− 0.0051**	− 0.0055**	− 0.0074
D_Ec_Growth	− 0.0125***	− 0.0142***	− 0.0092***	− 0.0101***	− 0.0077***	− 0.0050
D_Ec_Freedom	0.0058*	0.0022	0.0041**	0.0023	0.0043	0.0064
L_Priv_Exp	− 0.1948***	− 0.2122***	− 0.1867***	− 0.1655***	− 0.1689***	− 0.1677***
L_Gov_Exp	− 0.0246**	− 0.0160***	− 0.0174***	− 0.0167***	− 0.0256***	− 0.0390***
L_GG_Debt	0.0004*	0.0008***	0.0004***	0.0002	0.0002	0.0001
L_GG_Balance	− 0.0082***	− 0.0057***	− 0.0070***	− 0.0079***	− 0.0106***	− 0.0118***
L_Ec_Growth	− 0.0079**	− 0.0135***	− 0.0074***	− 0.0068***	− 0.0073***	− 0.0024
L_Ec_Freedom	0.0070**	0.0091***	0.0066***	0.0060***	0.0064***	0.0036**
Constant	0.0498	− 0.2355**	− 0.0402	0.0193	0.1065	0.4413***

"Mean" correspond to the mean estimates (PARDL), and "10th", "25th", "50th", "75th", and "90th" are the PQARDL estimates for the 10%, 25%, 50%, 75%, and 90% quantiles of the Private Health Expenditure conditional distribution. ***, **, and * denote statistical significance at 1%, 5%, and 10% levels, respectively

**Table 12:** Short-run and long-run impacts

Independent variables	Dependent variable (Priv_Exp)					
	PARDL model	PQARDL model				
		Quantiles				
	Mean	10th	25th	50th	75th	90th
*Short-run impacts*						
Gov_Exp	− 0.0781	− 0.0822***	− 0.0056	0.0007	0.0195	0.0148
GG_Debt	0.0022**	− 0.0002	0.0010	0.0012	0.0013*	0.0032
GG_Balance	− 0.0056*	− 0.0061	− 0.0059***	− 0.0051**	− 0.0055**	− 0.0074
Ec_Growth	− 0.0125***	− 0.0142***	− 0.0092***	− 0.0101***	− 0.0077***	− 0.0050
Ec_Freedom	0.0058*	0.0022	0.0041**	0.0023	0.0043	0.0064
*Long-run impacts*						
Gov_Exp	− 0.1262**	− 0.0755***	− 0.0933***	− 0.1007***	− 0.1515***	− 0.2327***
GG_Debt	0.0021*	0.0038***	0.0023***	0.0015	0.0010	0.0003
GG_Balance	− 0.0421***	− 0.0271***	− 0.0373***	− 0.0476***	− 0.0629***	− 0.0705***
Ec_Growth	− 0.0405***	− 0.0636***	− 0.0398***	− 0.0413***	− 0.0430***	− 0.0148
Ec_Freedom	0.0360***	0.0430***	0.0353***	0.0360***	0.0380***	0.0216**
*Speed of adjustment*						
ECM	− 0.1948***	− 0.2122***	− 0.1867***	− 0.1655***	− 0.1689***	− 0.1677***

"Mean" corresponds to the mean estimates (PARDL), and "10th", "25th", "50th", "75th", and "90th" are the PQARDL estimates for the 10%, 25%, 50%, 75%, and 90% quantiles of the Private conditional distribution. ***, **, and * denote statistical significance at 1%, 5%, and 10% levels, respectively

The results presented in Table 11 demonstrate several noteworthy findings. First, in the PARDL model, we observe that General Government Debt and Economic Freedom first differences have a positive impact on private healthcare expenditures (as enunciated in hypotheses h2.1 and H4), while Economic Growth and the General Budget Balance exhibit a negative effect (hypotheses h2.2 and H3). Furthermore, the lagged variables consistently display statistical significance, with GG_Debt and Ec_Freedom positively influencing private healthcare expenditures, whereas the remaining variables have a negative impact.

The pattern of quantile regression estimates aligns well with the findings obtained from the PARDL model, thus supporting the hypotheses of this study. The first difference coefficient of GG_Debt maintains the same sign but is statistically significant only at the 75th quantile, so hypothesis h2.1 is mainly verified at high levels of private health expenditures. Similarly, there is a notable agreement between PARDL and quantile regression results for D_GG_Balance and D_Ec_Growth. Additionally, the quantile estimates reveal a negative effect of D_Gov_Exp at the 10th quantile (as stated in hypothesis h1.1). Thus, there is evidence of a substitutability effect between public and private health expenditure only for countries with low levels of private health expenditure. On the other hand, D_Ec_Freedom shows a positive effect at the 25th quantile (hypothesis H4).

The estimates for lagged independent variables also exhibit high consistency between PARDL and quantile regression models, both in sign and significance. The exception to this consistency is L_GG_Debt, which is only significant for the bottom quantile, where private health spending is low, and households may find public health services unattractive (hypothesis h1.1). In addition, the PQARDL estimates show that the negative effects of government health expenditure and the general government balance are more pronounced for countries with a high level of private health expenditure, while the opposite pattern is true for economic growth.

**Fig. 3:**
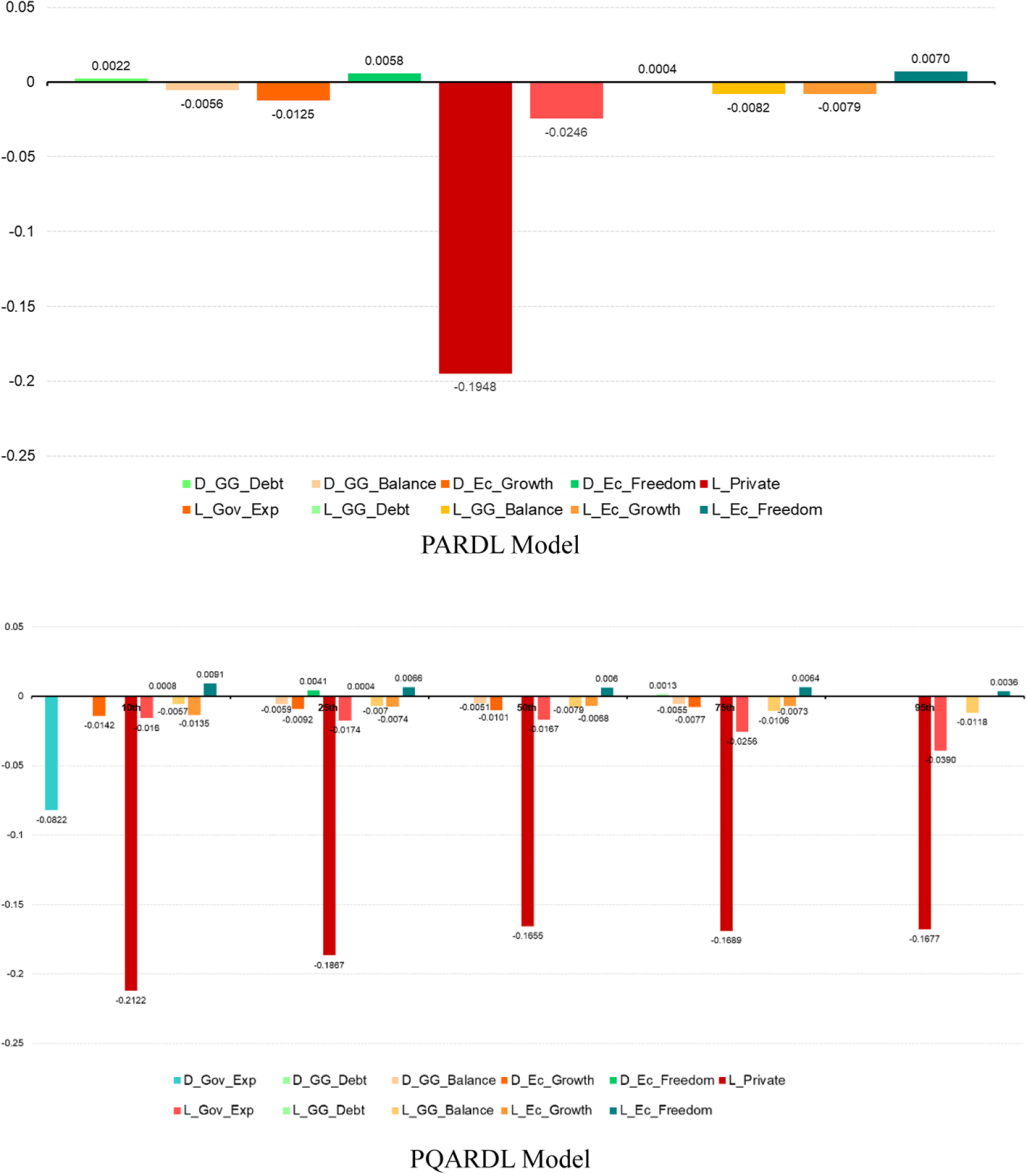
Graphical depiction of the impacts. The authors created this figure

Overall, these findings highlight the impact of various factors on household healthcare expenditures and emphasize the importance of considering both the short and long-run effects of covariates. The agreement between the PARDL and quantile regression results further strengthens the validity of the findings, providing valuable insights into the relationship between these variables. Indeed, Fig. 3 below shows the graphical depiction of the impacts of independent variables on the dependent variable in both the PARDL and PQARDL models.

After presenting the results from Table 11, this investigation can now examine the short- and long-run effects. Therefore, Table 12 below displays the results of the short-run and long-run effects in both the PARDL and PQARDL models.

**Fig. 4:**
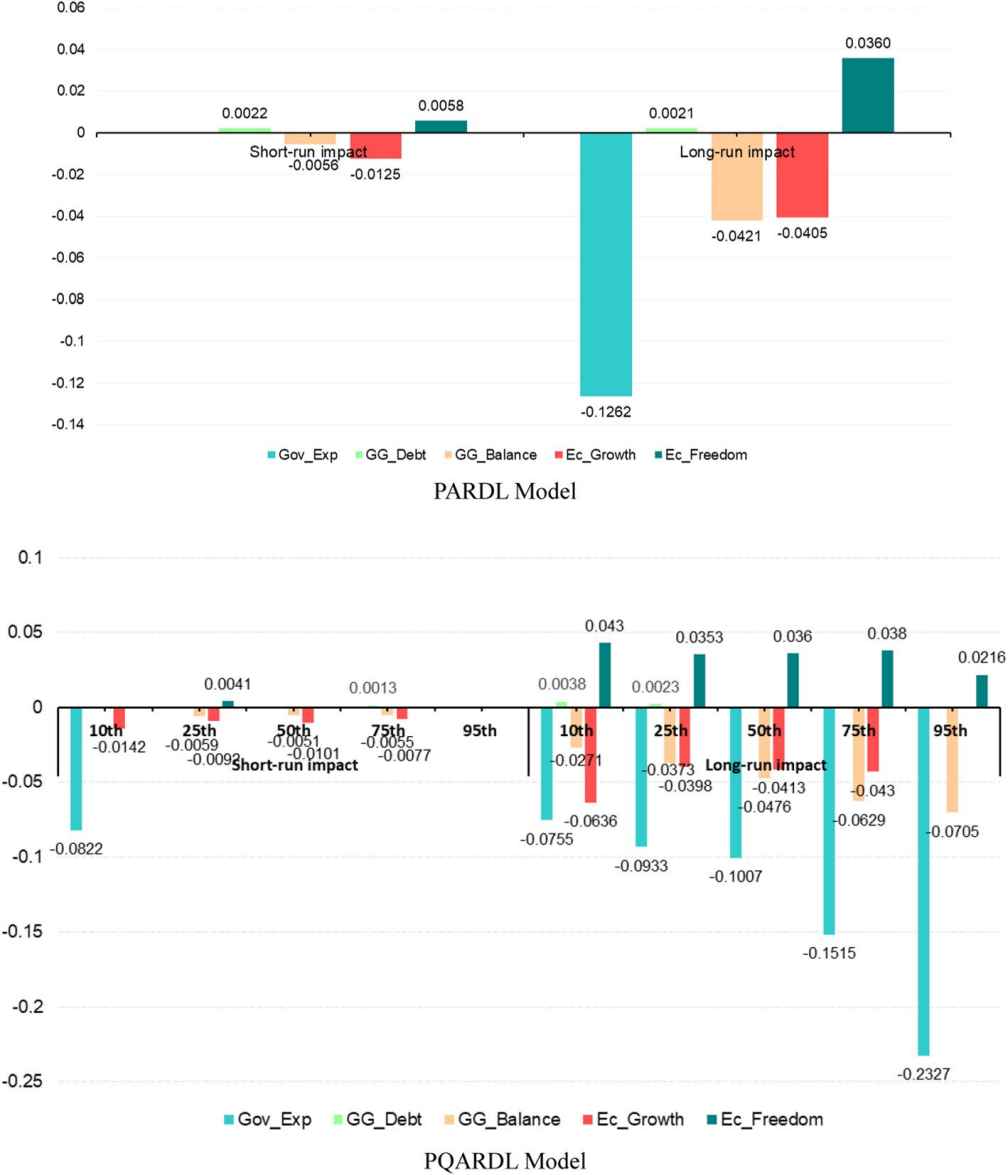
Graphical depiction of the short-and long-run impacts. The authors created this figure

Table 12 above shows high substitutability between private and public healthcare spending in the long run (hypothesis H1). In the mean regression, a 1% rise in public healthcare spending leads to a 0.1262% decrease in private expenditures. PQARDL estimates show that this substitutability is stronger in countries with high private health expenditures. The negative relationship between public and private healthcare spending is also noticeable in the short run for the lowest quantile. Another finding shows that private healthcare expenditures are income-inelastic: an increase in Economic Growth by 1% leads to a decrease in household private health spending as a fraction of GDP or an increase in private expenditures smaller than 1% (as postulated in hypothesis h3.1). This effect is mostly noticeable in the long run and for the lowest quantiles. However, it is also observable in the short run. The General Government Balance also has a negative effect on the dependent variable (hypothesis h2.2). However, unlike for Economic Growth, it is stronger in the highest quantiles. Both GG_Debt and EC_Freedom impact household healthcare expenditure positively (hypotheses h2.1 and H4), mostly in the long run and in the highest quantiles. The speed of adjustment coefficients implies that the dependent variable of this investigation returns to equilibrium after 5 and 6 years. Indeed, Fig. 4 below shows the graphical depiction of the short- and long-run impacts of independent variables on the dependent variable in both the PARDL and PQARDL models.

The following section will discuss the results, focusing on the relationship between the independent and dependent variables.

## Discussion

This section begins by presenting the main findings of this study. Then, it proceeds by discussing the current knowledge presented in the literature about health expenditures and the contribution of this research.

The main findings of this study are summarized in Table 12. Firstly, this investigation highlights the positive influence of government debt and economic freedom on private health expenditures. Although the short-run effects are less evident for each quantile of private expenditures, they exist at the sample mean. The long-run effects are more relevant and observable across the quantiles. The results suggest that an increase in government debt or broader economic freedom tends to be associated with higher private health expenditures. Nevertheless, the impact of government debt on private health expenditures is not statistically significant for levels of private health above the median.

Secondly, this investigation highlights the negative influence of government budget balance, health expenditures, and economic growth on private health expenditures. However, government expenditures have no significant short-run effect on average private health expenditure. As the government budget balance improves (for instance, it becomes less negative), as government expenditures increase, and with economic growth, there is a decrease in private health expenditures.

The macroeconomic analysis of the relationship between private health expenditures and macroeconomic and fiscal conditions is not well-explored in the literature, except for the relationship between health expenditures and economic growth. This relationship is extensively examined in different studies, and the general result is a positive correlation. Economic growth motivates health expenditure (Atems, 2019; Beylik et al., 2022; Bhat & Jain, 2004; Guisan & Arranz, 2003; Hitiris & Posnett, 1992; Ozyilmaz et al., 2022; Samadi & Homaie, 2013; Xu et al., 2011), and the principal driver for this relationship is capital accumulation, including human, physical, and knowledge capital. Considering that private health expenditures are a component of total health expenditures, private health expenditures are also expected to increase with economic growth (Beraldo et al., 2009). Another mechanism supporting these relationships is based on a Keynesian view that government expenditure is a policy instrument that contributes to economic growth.

Private health expenditures often display a negative correlation or substitutive effect with public health expenditures (Guisan & Arranz, 2003; Tuohy et al., 2004), although the nature of this relationship might not always be unequivocal (Xu et al., 2011). Reducing public spending on healthcare may cause people to look for alternative private providers to pay for people or in rationing activities. However, the extent and intensity of this change may depend on the type of health system financing. Financed by taxes, national health systems might be susceptible to governmental decisions and political cycles. Conversely, in social health insurance systems, direct governmental control over health expenditures is limited due to financing primarily relying on employment contributions. On the other hand, lower public spending may be due to increased patient cost-sharing. Still, because of low price elasticity, people may not substitute their utilization of public services for those offered by the private sector, which may also result in high prices (Braendle & Colombier, 2016).

Following the government decision on health expenditures just described, there is the government budget balance. When this balance is a surplus, there are no fiscal concerns. When this balance is negative, concerns about its change over time exist. Improving the fiscal balance is often achieved by reducing government spending. This decrease in public expenditure may occur in the health sector and affect the provision of health services. Alternatively, it may come from tax increases. The findings support the latter idea that fiscal consolidation reduces families' disposable income (hypothesis h2.2(ii)), leading to reduced private healthcare expenditure.

From an economic theory standpoint, substantial government debt arises from the accumulation of prior negative government budgets and interest payments. As countries face increasing debts and increasing international pressure for fiscal sustainability, it may be a political choice to reduce public health expenditure by decreasing the supply in terms of quantity or quality, which is compensated by increasing private expenditures in the private sector (Achdut, 2019; Bock et al., 2014; Wolf & Toebes, 2016; You & Kobayashi, 2011). This correlation might be more pronounced when government debt hampers economic growth, diminishes employment levels, thereby impacting funding for social health insurance schemes, and reduces tax revenues, necessitating a reduction in public expenditures. On the other hand, international pressure and IMF bailout programs may force governments, formally or informally, to decrease public health expenditures, as has happened in Greece and Portugal (Palasca & Jaba, 2015).

When the public health sector faces constraints, coupled with increased economic freedom, it can create room to expand the private healthcare sector. This includes healthcare provision, pharmacies, alternative and complementary medicines, the medical devices sector, and innovative health technology (Basu et al., 2012; Gasmi & Benlamri, 2022; Roberts & Olson, 2013; Wode et al., 2019). There is no evidence for this relationship in the literature. However, one may infer that since (i) economic growth goes together with economic freedom (Brkić, 2020; Haan & Sturm, 2000; Scully, 2002) and (ii) economic growth is related to the increase in health expenditures, including private health expenditures (Aydan et al., 2021), then it may be expected that economic freedom will be correlated with private health expenditures depending on the public investment in healthcare and the quality of public healthcare. Despite the potential improvement in well-being associated with this economic freedom (Stroup, 2007), private provision of healthcare services may raise equity concerns and endanger UHC. Moreover, competition between providers is strongly regulated in European countries or limited or non-existent as in Japan (Edwards, 2018), and the effects on health and healthcare quality are not guaranteed (Siciliani et al., 2022).

The empirical results of this investigation have shown that macroeconomic and fiscal conditions impact private health expenditures. First, government debt appears to increase private health expenditures, even in periods without an economic or financial crisis, as occurred during the 2010 European debt crisis. The debt pressure on the government from international institutions or the Maastricht agreement forces them to opt for policies that clearly penalize public health services, driving people to seek healthcare in the private sector. This finding adds to the seed idea that safeguarding social and health spending protects vulnerable populations who do not have the means to access private healthcare.

A novel contribution of this research shows that economic freedom is associated with increasing private health expenditures. This may be happening due to a broader healthcare offering from the private sector, although it is highly regulated in most European countries. A range of health-related goods and services can be accessed in the market through private providers, such as pharmaceuticals, medical exams, medical devices, health technology, new treatments, and alternative and complementary medicine.

This investigation found that an improvement in the government budget balance and a decrease in government health expenditures result in higher private health expenditures. These findings follow the previous findings of this investigation regarding the relationship between government debt and private health expenditures. An enhanced government budget balance can be achieved through increased tax collection, expenditure reduction, or a combination. Periods of economic expansion, marked by high employment rates, wages, and income, tend to yield increased tax revenues, leading to elevated private health expenditures. Reducing government spending is also likely to occur during periods of fiscal adjustment or international pressures for sustainable fiscal conditions. It usually affects the health sector, and individuals often compensate by choosing to access healthcare services and goods in the private sector.

To sum up, this study has demonstrated the influence of fiscal conditions on private health expenditures, which has not been fully explored in previous studies (Fan & Savedoff, 2014). Indeed, there are situations where external factors pressure fiscal sustainability, often leading to elevated household spending on healthcare. These findings raise concerns about universal health coverage, the prevalence of catastrophic health expenditures, and equitable access to healthcare in OECD countries.

## Conclusion

This study addresses the following general research question: What is the link between public finances and households' consumption of private health goods and services? Considering the vast diversity of health systems worldwide, this investigation focused on countries with a high level of similarity. The main hypotheses of this study that can be tested are: (i) there is substitutability between public and private health services, (ii) the state of public finances impacts the provision of private health services, (iii) economic growth improves public finances, enabling the provision of public health services (which, in turn, decreases private health services as a percentage of GDP), and (iv) the preference for economic freedom leads households to seek more private health services.

The research focuses on twenty-six European countries that were members of the OECD from 1995 to 2019, employing a variety of econometric techniques such as Panel Autoregressive Distributed Lag (PARDL), Panel Quantile Autoregressive Distributed Lag (PQARDL), and the panel Granger non-causality test proposed by Dumitrescu and Hurlin (2012). These techniques allow us to explore multiple dimensions of the relationship, including short and long-run impacts and differences between quantiles. Additionally, Granger's non-causality tests were employed to support the main analysis.

The PARDL and PQARDL estimations revealed an error correction mechanism (ECM) with a negative sign, falling within the range of − 1 and 0, and were statistically significant. These results confirm the presence of cointegration among variables, indicating an improvement in the analysis by decomposing the total impacts into their short- and long-run components.

Testing the hypotheses related to the research question led us to conclude that (i) government debt and economic freedom have a positive impact on private health expenditures, and (ii) the government budget balance, government health expenditures, and economic growth have a negative impact on private health expenditures. These findings support the notion that fiscal conditions will likely affect private health expenditures. In short, macroeconomic and fiscal conditions affect private health expenditures, raising concerns about the equity and financial protection aspects of universal health coverage in OECD countries.

From the point of view of the policy implications of this study, policymakers could adopt a comprehensive approach to healthcare, recognizing that health is a semi-public good and that the substitutability between public and private health services can lead to suboptimal outcomes. While fiscal consolidation is necessary for the macro economy, it should be accompanied by targeted policy measures to prevent the deterioration of health among disadvantaged individuals and ensure equitable access to healthcare, regardless of fiscal conditions. Gradual and non-shocking approaches to fiscal consolidation, tailored to each country's conditions and context, strengthen long-term effects. Economic freedom should be advanced with safety nets for vulnerable citizens, while regulatory oversight must be strengthened in the expanding private healthcare sector. Emphasis on preventive healthcare, data-driven decision-making, and collaboration with civil society will collectively enable a balanced, equitable, and effective healthcare system that aligns with economic growth and social well-being.

The study's findings, while insightful, should be viewed within the context of certain limitations. The focus on a select group of developed European countries within the OECD restricts the generalizability of the results to a broader global context. Furthermore, while the decomposition of impacts into short- and long-run components enhances the analysis, a longer time frame could provide a more robust understanding of the relationship between macroeconomic and fiscal conditions and private health expenditures. It is essential to exercise caution when interpreting the results, as inherent limitations in empirical research, including the endogeneity of variables, potential omitted variable bias, and the risk of oversimplified models, may impact the accuracy and applicability of the conclusions. As such, future research should consider a more diverse sample and address potential methodological challenges to provide a more comprehensive understanding of the intricate dynamics in healthcare financing and its implications for public health outcomes.

The study's implications extend to several areas for future research and policy development. First, acknowledging the potential for asymmetrical effects, researchers could explore the nonlinear responses of private health expenditures to positive and negative changes in their determinants, enriching the understanding of these complex relationships. Second, to enhance the robustness and applicability of findings, extending the analysis to encompass middle and low-income countries would provide valuable insights into the diverse global health landscape. Third, policymakers should focus on crafting strategies that safeguard healthcare access irrespective of fiscal conditions, ensuring vulnerable populations are shielded from health deterioration. Fourth, to determine and understand the optimal mix of private and public health expenditures that enhance population health and mitigate health care inequities. Fifth, with a longer timeframe, a comparative analysis between the post-great recession and pre-recession periods could illuminate the impact of economic shocks on healthcare financing. Lastly, an essential avenue for research lies in investigating the correlation between fiscal situations and catastrophic health expenditures, contributing to the development of effective strategies for financial protection in healthcare systems.

To end this conclusion, a comment about the contribution of this work to Sustainable Development Goals (SDGs), particularly Goal 3: Good Health and Well-being, and Goal 10: Reduced Inequalities. This study sheds light on the relationship between macroeconomic and fiscal conditions and private health expenditures, crucial for informed policymaking to improve healthcare accessibility and affordability. This aligns with Goal 3's targets, ensuring universal access to quality healthcare services and achieving financial protection against health-related expenditures. Moreover, the emphasis of this investigation on preventive healthcare and equitable access, regardless of fiscal conditions, directly promotes overall health and well-being. Additionally, this research addresses inequalities in healthcare access by highlighting disparities resulting from fluctuations in macroeconomic and fiscal conditions. By emphasizing targeted policy measures to prevent health deterioration among disadvantaged individuals and strengthening regulatory oversight in the private healthcare sector, we contribute to Goal 10's aim of reducing inequalities within and among countries. This investigation provides valuable insights into healthcare financing dynamics, contributing to the global effort towards Sustainable Development Goals 3 and 10. By tackling the root causes of healthcare inequalities and promoting equitable healthcare systems, this research supports sustainable development and enhances the well-being of populations worldwide.

## Data Availability

Data is available from the corresponding author on a reasonable request.
